# First report of emerging fungal pathogens of *Cordyceps militaris* in Vietnam

**DOI:** 10.1038/s41598-023-43951-9

**Published:** 2023-10-17

**Authors:** Trung Thanh Nguyen, Thi Nguyen-Gia Le, Thuan Huy Nguyen

**Affiliations:** Center for Pharmaceutical Biotechnology, School of Medicine and Pharmacy, Duy Tan University, Danang, 550000 Vietnam

**Keywords:** Microbiology, Molecular biology

## Abstract

Cultivation of *Cordyceps militaris*, a valuable medicinal and edible fungus, has dramatically increased in Vietnam since 2010. During industrial production, parasitic white molds were found to infect the mycelia and fruiting bodies of *C. militaris* causing significant quality and yield losses. Two different fungal strains were obtained from the mycelia and fruiting bodies of *C. militaris* in Danang mushroom farms and were characterized by morphological and multiple DNA markers analysis. The sequence alignment of ITS, LSU and *rpb2* markers revealed that the pathogens are related to the type species *Lecanicillium coprophilum* and *Calcarisporium cordycipiticola* with more than 99% sequence identities. The growth characteristics and pathogenic activities of the two isolated species on their host *C. militaris* were also investigated. The phylogenetic analysis based on the ITS sequences showed that *L. coprophilum* WF2611 is closer to its host *C. militaris* than *C. cordycipiticola* NT1504. To our knowledge, this is the first worldwide report of *C. militaris* infected by *L. coprophilum* which would be an useful information on prevention and control of the disease and be helpful for the industrial cultivation of *C. militaris*.

## Introduction

*Cordyceps militaris* (L.) Fr., one of the most valued edible and medicinal fungi, has long been used as an herbal drug and tonic in China, Korea, Japan and other East Asian countries due to their high content of bioactive compounds beneficial to human health^1, 2^. The bioactive constituents and pharmaceutical properties of *C. militaris* have similar to the wild-type *Ophiocordyceps sinensis* (formerly known as *Cordyceps sinensis*) and are widely used as a substitute for *O. sinensis* in health supplements^3–5^. Compared to *O. sinensis*, the artificial cultivation of *C. militaris* was easier and successfully archived in the early 1980s^6^. In addition, *C. militaris* can easily grow and produce fruiting bodies in solid and liquid media with a variety of carbon and nitrogen sources^1^. The cultivation of *C. militaris* has been widespread in China, Japan, Korea, Thailand and Vietnam. It is estimated that the annual value of production of *C. militaris* in China only is approximately 10 billion CNY (about 1.57 billion $ US)^7^.

During the large-scale production of *C. militaris* in China, fungal diseases frequently occur. The pathogens were first identified as *Calcarisporium cordycipiticola* sp. nov., *Tricothecium crotocinigenum*, *Fusarium sp., Schizophyllum commune, Trichoderma harzianum, Purpureocillium lilacinum, Ustilaginoidea virens, Clonostachys rosea, T. ovalisporum, Penicillium expansum, Aspergillus oryzae* and *A. niger*^8–10^. Among them, *C. cordycipiticola* is the most serious pathogen causing infectious diseases on fruiting bodies of *C. militaris*. The morphological characteristics and pathogenic mechanism of *C. cordycipiticola* on its host have been reported in detail, however, little information on the prevention and control of the disease is suggested^11, 12^.

Recently, the artificial cultivation of *C. militaris* in Vietnam has dramatically increased and a similar situation of fungal diseases has also been recognized. Among them, pathogens with white cottony colonies were often found to infect the mycelia and fruiting bodies of *C. militaris* causing significant quality and yield losses. However, no report for infectious mycoparasite of *C. militaris* in Vietnam has been published yet. Therefore, the aims of this study were to isolate and characterize the mycoparasites infected with *C. militaris* in Vietnam. The fungal pathogens isolated in this study were identified by multiple DNA markers including two markers of nuclear ribosomal regions (ITS—Internal Transcribed Spacer and LSU—28S nuclear ribosomal large subunit rRNA gene) and one protein coding sequence (*rpb2* gene coding for the second largest subunit of RNA polymerase II). The morphological characteristics and the pathogenic activities of the fungal pathogens were then identified. Furthermore, the genetic relationships between the parasites and their host *C. militaris* were also investigated.

## Results

### Strain isolation and morphological observation

**Figure 1:**
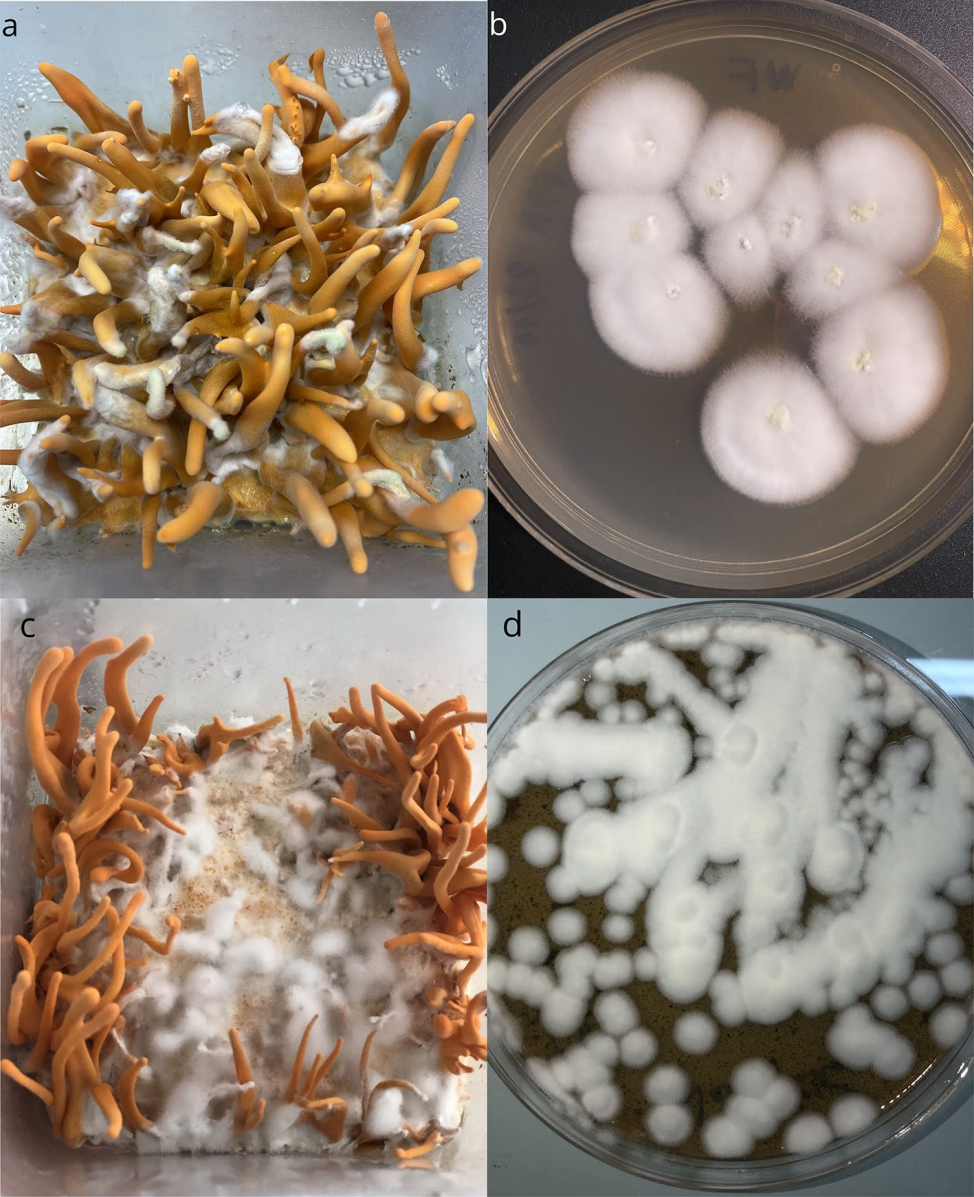
White mold pathogens on mycelia and fruiting bodies of *C. militaris* and colonies of isolated strains on PDA medium at 25 °C after 7 days. Strain WF2611 (**a**,**b**) and strain NT1504 (**c**,**d**).

In this study, two different parasitic fungi with white mycelia forming a cottony layer on mycelia and fruiting bodies of *C. militaris* were isolated and serially plated onto PDA-Petri dishes to get the pure strains named WF2611 and NT1504, respectively (Fig. 1). Colonies of strain WF2611 were creamy-white, cottony, light yellow that slowly grown on PDA and reached 10–13 mm diameter at 25 °C after 7 days (Fig. 1a,b). However, colonies of strain NT1504 were pure-white, cottony that grown faster but reached a smaller diameter of 3–5 mm at 25 °C after 7 days (Fig. 1c,d).

**Figure 2:**
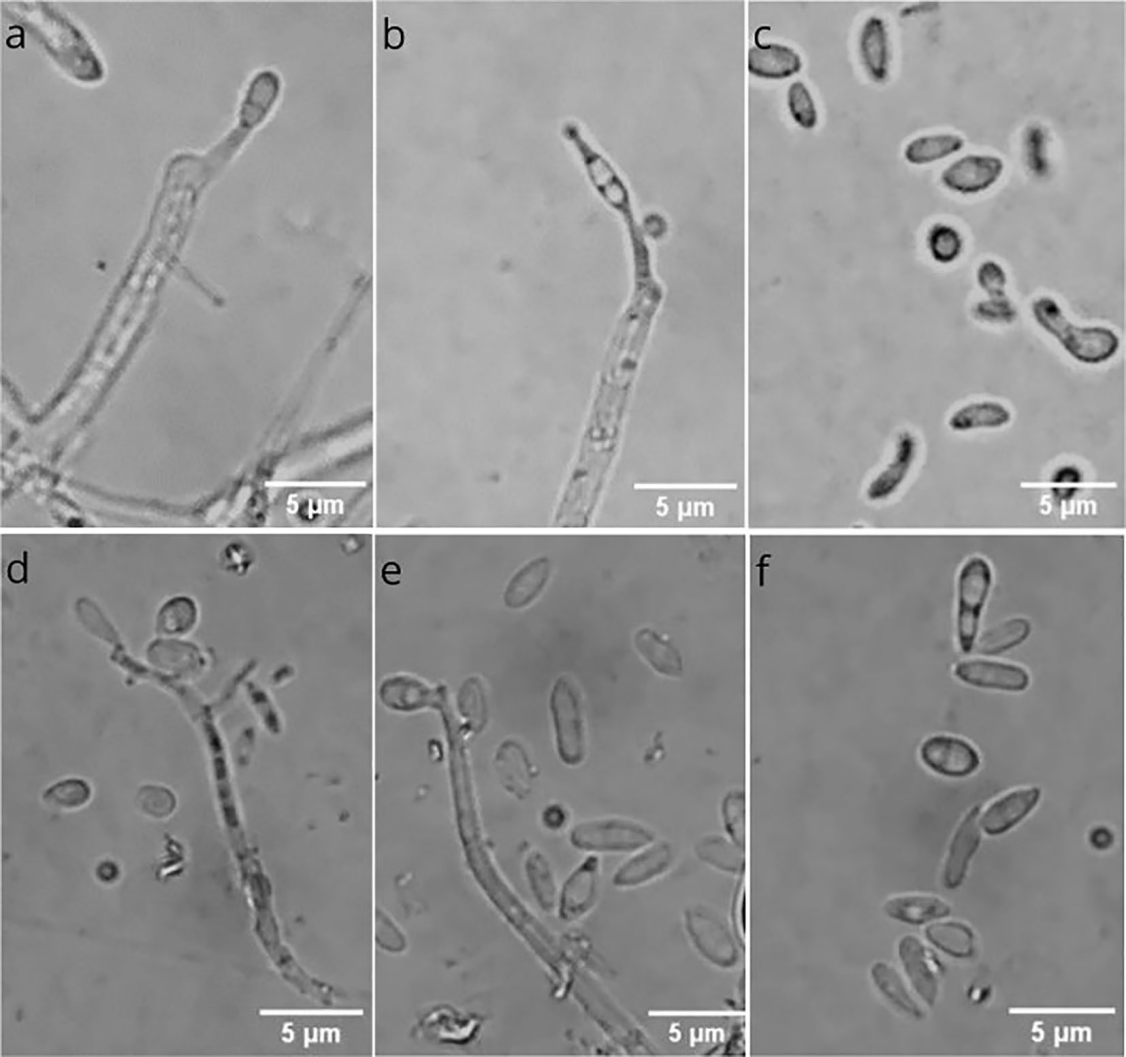
Microscopic analyses of isolated fungal strain WF2611 (**a**–**c**) and NT1504 (**d**–**f**). Conidiophores with conidia (**a**,**b**,**d**,**e**); conidia (**c**,**f**). Bars: 5 µm.

The microscopic analyses showed the conidiophores and conidia of the two isolated strains (Fig. 2). Conidiophores of strain WF2611 arose from aerial hyphae and moderately branched. Conidia (2–5 × 1–2 µm) aggregated to the apex of the conidiophores with shapes ranging from ellipsoidal to falcate or fusiform with round ends (Fig. 2a–c). For strain NT1504, there has more conidia formed around conidiophores and the conidia have different shapes but most of them are ovoid or fusiform with a size of 2–4 × 1–1.5 µm (Fig. 2d–f).

The pathogenicity test revealed that white cottony colonies of both strains developed on all five inoculated at any stage of growth, while the controls remained symptomless. The pathogens first invaded the mycelia, then proliferated on the surface of fruiting bodies of *C. militaris*. Strain NT1504 invaded the host *C. militaris* faster than strain WF2611. After 15–30 days, the white mold pathogens covered completely the fruiting bodies and the colour of the surface of fruiting bodies turned into grey and died. The morphological characteristics of white mold pathogens on inoculated cultures were identical to strains observed on the original infected fruiting bodies.

### Strain identification

**Figure 3:**
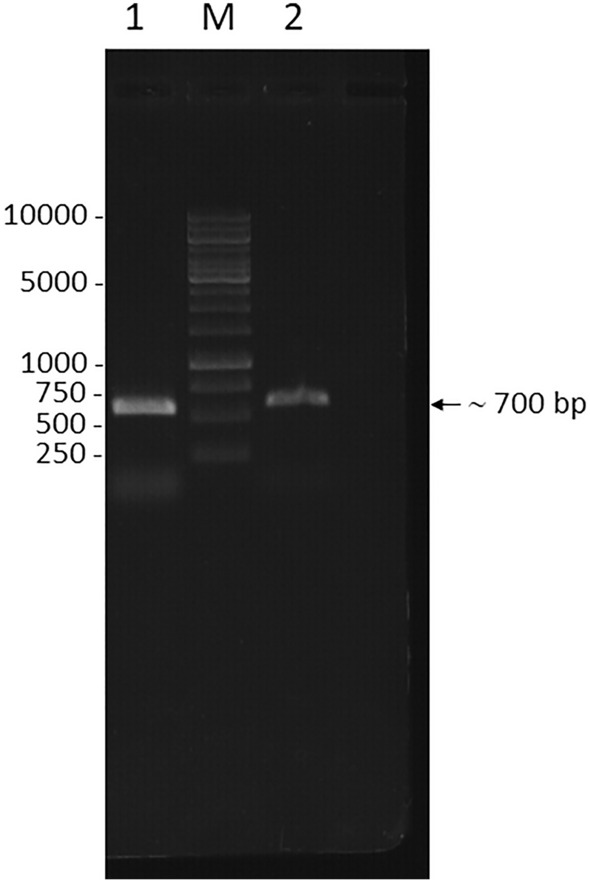
Electrophoresis of the PCR products containing the complete ITS region of strain NT1504 (1) and WF2611 (2) on 0.8% agarose gel. M: 1 kb DNA ladder.

In order to identify the fungal pathogens, we amplified the complete ITS region of approximately 700 bp containing ITS1-5.8S-ITS2 from the DNA genome of isolated strains by using the primer pairs ITS5-F/ITS4-R (Fig. 3). Fragments of approximately 1100 bp containing 28S rDNA (LSU) from both isolated strains were also amplified by the primer pairs LROR-F/LR6-R. Finally, the *rpb2* gene of approximately 1200 bp from both strains were amplified by the primer pairs RPB2-5F/RPB2-7cR. Those PCR products were then purified and sequenced by one direction for the sequence analysis and species identification.

The ITS, LSU and *rpb2* sequences obtained from sequencing data in this study were aligned with reference sequences by Basic Local Alignment Search Tool (BLAST) in Genbank. Firstly, the ITS sequence alignment showed that strain WF2611 and NT1504 exhibited 99.82% and 99.43% similarity with *Lecanicillium coprophilum* CGMCC 3.18986 (NR_163303) and *Calcarisporium cordycipiticola* MFLUCC 15–0685 (NR_144864), respectively. Sequences of ITS region of both strains were then deposited to GenBank with the accession numbers of OQ625887 and OQ625889, respectively. Similarly, the LSU sequences of strain WF2611 and NT1504 showed 99.89% and 99.42% similarity with *L. coprophilum* CGMCC 3.18986 (NG_067818) and *C. cordycipiticola* CGMCC 3.17905 (NG_067538), respectively. The GenBank accession numbers for the LSU sequences of WF2611 and NT1504 strains were OR272054 and OR272052, respectively. Finally, the *rpb2* sequence alignment showed that strain WF2611 exhibited 99.16% and 100% similarity in nucleotide and protein sequences with *L. coprophilum* TBS415 (MH177624), respectively (Fig. S1). Although the *rpb2* nucleotide sequence of strain NT1504 exhibited 99.40% similarity with *C. coprophilum* CGMCC 3.17940 (KX442607), the protein sequence alignment showed a reading frame shift near the 3’-end of the sequence due to the insertion of one Adenine (A) at nucleotide position 958 (Fig. S2). The sequences of *rpb2* gene from strain WF2611 and NT1504 were deposited on GenBank with the aceession numbers OR500227 and OR500228, respectively. Based on those multiple markers analysis, we introduced the newly isolated strains as *Lecanicillium coprophilum* WF2611 and *Calcarisporium cordycipiticola* NT1504.

### Phylogenetic analysis

**Figure 4:**
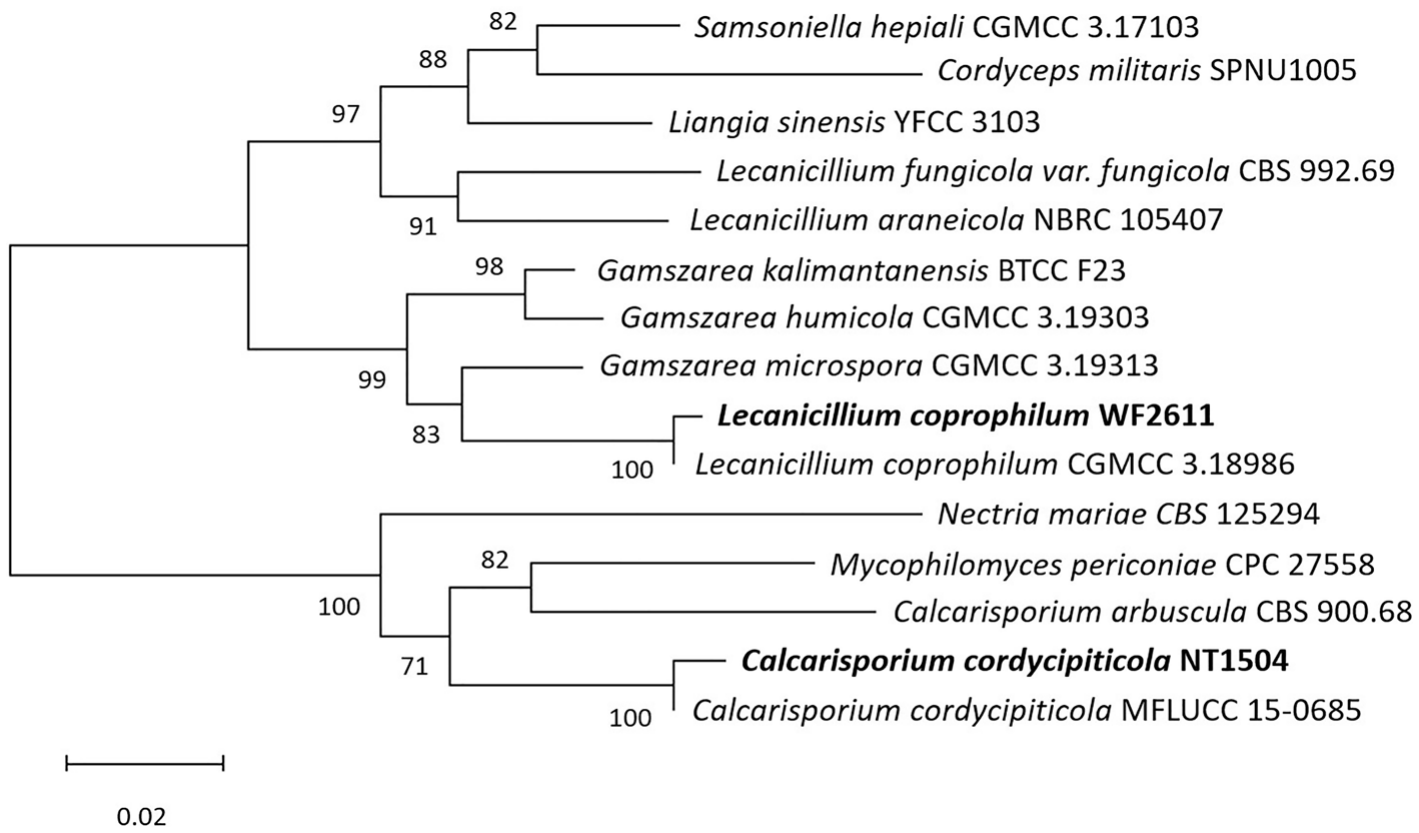
Phylogenetic tree derived from Neighbour-joining analysis based on ITS sequences of the isolated strains and related taxa. Numbers at the branches represent bootstrap percentages. The newly isolated strains in this study were shown in bold.

The ITS sequences from *L. coprophilum* WF2611, *C. cordycipiticola* NT1504 and related species downloaded from GenBank (Table 1) were aligned by Mega11 using the ClustalW algorithm. As shown in Fig. 4, the newly isolated strain *C. cordycipiticola* NT1504 formed a clade with *Calcarisporium arbuscula* CBS 900.68 and *Mycophilomyces periconiae* CPC 27,558 with bootstrap support of 71%. For the strain *L. coprophilum* WF2611, it formed a clade with *Gamszarea microspora* CGMCC 3.19313 and other *Gamszarea* species with bootstrap support of 83% and 99%, respectively. However, species of *L. coprophilum* formed a cluster distinct from other *Lecanicillium* species with strong bootstrap support of 97%. Furthermore, the phylogenetic tree in Fig. 4 also revealed that *L. coprophilum* WF2611 is phylogenetically closer to its host *C. militaris* than *C. cordycipiticola* NT1504.

**Table 1:** Species information and GenBank accession number for ITS sequences used for phylogenetic analyses.

Species	Strain no	GenBank accession no
*Lecanicillium coprophilum*	CGMCC 3.18986	NR_163303
*Lecanicillium coprophilum*	WF2611	OQ625887 (This study)
*Lecanicillium araneicola*	NBRC 105,407	NR_121208
*Lecanicillium fungicola var. fungicola*	CBS 992.69	NR_119653
*Gamszarea kalimantanensis*	BTCC F23	NR_121200
*Gamszarea humicola*	CGMCC 3.19303	NR_172830
*Gamszarea microspora*	CGMCC 3.19313	NR_172832
*Mycophilomyces periconiae*	CPC 27,558	NR_154209
*Liangia sinensis*	YFCC 3103	NR_173887
*Nectria mariae*	CBS 125,294	NR_160238
*Samsoniella hepiali*	CGMCC 3.17103	NR_160318
*Cordyceps militaris*	SPNU1005	KY407777
*Calcarisporium arbuscula*	CBS 900.68	KT945003
*Calcarisporium cordycipiticola*	MFLUCC 15–0685	NR_144864
*Calcarisporium cordycipiticola*	NT1504	OQ625889 (This study)
*Purpureocillium lilacinum*	NRRL 895	NR_165946

## Discussion

The large-scale prodution of *C. militaris* has been grown rapidly in Vietnam from 2010 and the problems of fungal diseases causing yield loss occurred very often, however, no information about the causative agents or the control methods have been reported until now. Therefore, our study was the first report of emerging fungal pathogens of *C. militaris* in Vietnam. Although there are many molecular markers used for barcoding, this study used ITS, LSU and *rpb2* markers due to their advantages and their widely used for the identification of fungal samples. Firstly, the complete ITS region is commonly used for the classification of isolated fungal strains at the species level^13^. In addition, the available of 15.972 curated complete ITS sequences with correct taxonomic names of fungal type specimens from the ITS RefSeq Targeted Loci project provides great references for us to classify the isolates (https://www.ncbi.nlm.nih.gov/bioproject/PRJNA177353/). Furthermore, the ITS5/ITS4 are also the standard primer pairs used to amplify the complete ITS region and there has 8.421 available ITS sequences amplified by these primer pairs in the GeneBank database^13^. The LSU sequence amplified by using the primer pairs LROR-F and LR6-R is normally used to identify fungi at higher taxonomic levels such as family or genera. In addition, the combination of LSU and ITS regions can also be valuable for the identification of fungi at the species level^14^. Using the ITS or LSU marker alone for fungal identification at species level might not suffice for some genera of phylum Ascomycota, including *Aspergillus*, *Penicillium* as well as *Lecanicillium*^15^. Therefore, in this study we used an protein-coding gene named *rpb2* as an additional marker to deal with that problem. Protein-coding genes used for fungal identification have some advantages such as they occur as single copy in fungi and they have intron regions in their sequences, which sometimes evolve at a faster rate compared to ITS or LSU^15^. The nearly 100% similarity in ITS, LSU and *rpb2* sequences of the isolated fungal samples and references from GenBank revealed that those markers are very suitable for the classification of microfungi at the species level.

In this study, two different fungicolous fungi were isolated from the fruiting bodies of infected *C. militaris*. Interestingly, one of them belongs to *C. cordycipiticola* that has firstly been isolated and recognized as an important fungal pathogen of *C. militaris* in China^8^. Biological characteristics and pathogenic mechanism of this fungicolous fungus on its host have been identified^16^. Furthermore, *C. militaris* is thought to be the only host of *C. cordycipiticola*^11^. In our work, the microscopic and growth characteristics of the newly isolated strain *C. cordycipiticola* NT1504 share similarities with previous reports of Sun et al.^8^. The pathogenicity test in this study also confirmed that *C. cordycipiticola* NT1504 is a strong fungal pathogen that can invade and reduce the production of *C. militaris* in a short period of time. The ability to produce a large number of conidia could be the reason for the rapid invasion of this pathogen^16^. Furthermore, the artificial cultivation of *C. militaris* in Vietnam must use air conditioners to maintain the suitable temperature for the growth of mushroom. This could disperse more fungal conidia into the air and thus accelerate the infection process.

The other fungal pathogen isolated in this work was identified as *L. coprophilum*. The species of *Lecanicillium* are recognized as mycoparasites of various arthropods, nematodes, and other fungi. Among them, *L. coprophilum* is first isolated and characterized from feces of *Marmota monax*, a species belonging to the group of large ground squirrels known as marmots. *L. coprophilum* differs from other *Lecanicillium* species by the morphological characteristics of conidiogenous cells, conidia, dictyochlamydospores and swollen hyphae^17^. Microscopic morphology of conidia and conidiophores of strain *L. coprophilum* WF2611 isolated in this study shares the same morphological characteristics with reports of Su et al.^17^. Many *Lecanicillium* species are entomopathogenic or fungicolous fungi, however, to our knowledge this is the first worldwide report that *L. coprophilum* is a mycoparasite of *C. militaris*.

The invasion process of a fungal parasite to its host depends on many factors. Among them, the close relationship between invader and its host could affect the effectiveness of the infection process. The phylogenetic analysis in this study indicated that *L. coprophilum* WF2611 is closer to its host *C. militaris* than *C. cordycipiticola* NT1504. This result is supported by the fact that the genera *Lecanicillium* and *Cordyceps* belong to the same family Cordycipitaceae, however, the genus *Calcarisporium* belongs to the family Calcarisporiaceae. In the order Hypocreales, Calcarisporiaceae and Cordycipitaceae are sister families^18^. The closer relationship between *L. coprophilum* WF2611 and its host *C. militaris* could be an advantageous characteristic for the effective interaction and invasion of the pathogen. Until now, no information about the pathogenicities or the infection mechanisms that *L. coprophilum* uses to invade the host *C. militaris* has been reported. Therefore, the pathogenicities, infection process, toxicities, host specificity, as well as the methods for prevention of this parasite should be further investigated.

## Methods

### Sample collection and culture conditions

Samples were collected from infected *C. militaris* growing on sterilized brown rice at Vinseed Biotechnology Co. Ltd. (N16^o^ 01′ 37.64′′, E108^o^ 22′ 06.95′′), Danang and some other farms in the central and southern regions of Vietnam in October 2022. Infected *C. militaris* fruiting bodies were aseptically transferred onto Potato Dextro Agar (PDA) in Petri dishes and incubated at 25 °C for 7 days. All samples were serially plated onto PDA in Petri dishes and incubated at 25 °C to get the pure strains. The ex-type living cultures were deposited in the Center for Pharmaceutical Biotechnology, School of Medicine and Pharmacy, Duy Tan University, Danang, Vietnam.

### Morphological observation and pathogenicity test

All isolates were grown on PDA in Petri dishes and incubated at 25 °C for 7 days in darkness. The hyphal elongation was measured each day. Colony morphology and microscopic characteristics were examined, measured and photographed after incubation for 7 days. Microscopic observations were made from preparations mounted in 50% lactic acid. The structure and morphology of conidiophores were described from conidiophores obtained from the edge of conidiogenous pustules or fascicles of mature conidia.

The pathogenicity test was performed by gently dusting conidia of fungal pathogen onto five healthy fruiting bodies cultures of *C. militaris* and then growing under a condition of 25 °C, 85% humidity. Five noninoculated fruiting body cultures were used as controls^9^.

### DNA extraction, PCR amplification, and DNA sequencing

Fresh mycelia (30 mg) were harvested from a 7-day-old plate and put into 1.5 mL Eppendorf tubes for genomic DNA extraction. Genomic DNA was extracted following the protocol of Plant genomic DNA extraction mini kit (Favorgen, Taiwan). In order to identify the fungal strains, three different molecular markers were amplified by polymerase chain reaction (PCR) and then sequenced. Firstly, the rDNA fragment containing ITS1-5.8S-ITS2 of approximately 700 bp was amplified using the primer pairs ITS5-F (5’-GGAAGTAAAGTCGTAACAAGG-3’) and ITS4-R (5’-TCCTCCGCTTATTGATATGC-3’)^15^. For the large subunit (LSU) ribosomal DNA (rDNA) marker based on the partial 28S rDNA, we used the primer pairs LROR-F (5’-ACCCGCTGAACTTAAGC-3’) and LR6-R (5’-CGCCAGTTCTGCTTACC-3’)^15^ to amplify a fragment of approximately 1100 bp. Lastly, the *rpb2* gene (approximately 1200 bp in length) encoding the second largest subunit of RNA polymerase II was amplified using the primer pairs RPB2-5F (5’- GAYGAYMGWGATCAYTTYGG-3’) and RPB2-7cR (5’- CCCATRGCTTGYTTRCCCAT-3’)^19^. Each amplification reaction was performed in 25 µL reaction volume containing 2.0 µL of genomic DNA solution, 1.0 µL of each forward and reverse primers (100 pM/µL), 12.5 µL of 2 × DreamTaq PCR Master Mix (Thermo Fisher Scientific Baltics UAB, Lithuania) and 8.5 µL of ddH_2_O. The PCR parameters: 95 °C for 3 min; followed by 30 cycles at 95 °C for 30 s, 56 °C for 1 min, 72 °C for 1 min; and a final extension at 72 °C for 10 min were applied for the ITS and *rpb2* markers. For the LSU marker, the annealing temperature parameter was 52 °C. The PCR products were then run on 0.8% agarose gel for 30 min and the DNA bands of each markers were cut and purified by GeneJET gel extraction kit (Thermo Fisher Scientific Baltics UAB, Lithuania). The purified PCR products were then sequenced by 1st BASE (Malaysia).

### Phylogenetic analysis

The nucleotide sequences obtained from sequencing were compared to the references of ITS sequences from type material retrieved from BLAST database for ITS RefSeq at BioProject page (https://www.ncbi.nlm.nih.gov/bioproject/PRJNA177353/) (NCBI, USA). The ITS sequence of isolates and related species from GenBank (Table 1) were aligned using ClustalW method. The phylogenetic tree was reconstructed using the neighbor-joining method and the reliability of the tree was estimated by the bootstrap method. All the evolutionary analyses were conducted in MEGA 11^20^.

### Supplementary Information

Supplementary Figure S1.

Supplementary Figure S2.

Supplementary Legends.

## Data Availability

The DNA marker sequences of *L. coprophilum* WF2611 and *C. cordycipiticola* NT1504 are available on GenBank databases with the GenBank accession numbers: OQ625887 and OQ625889 for the ITS marker; OR272054 and OR272052 for the LSU marker; OR500227 and OR500228 for the *rpb2* marker, respectively. Other data and analyses generated during the current study are included in this published article and in the supplementary information.
