# Supplementary figures and images for: First report of emerging fungal pathogens of Cordyceps militaris in Vietnam
Source: Sci Rep. 2023 Oct 17;13:17669. doi: 10.1038/s41598-023-43951-9 (PMC10582018; doi:10.1038/s41598-023-43951-9)

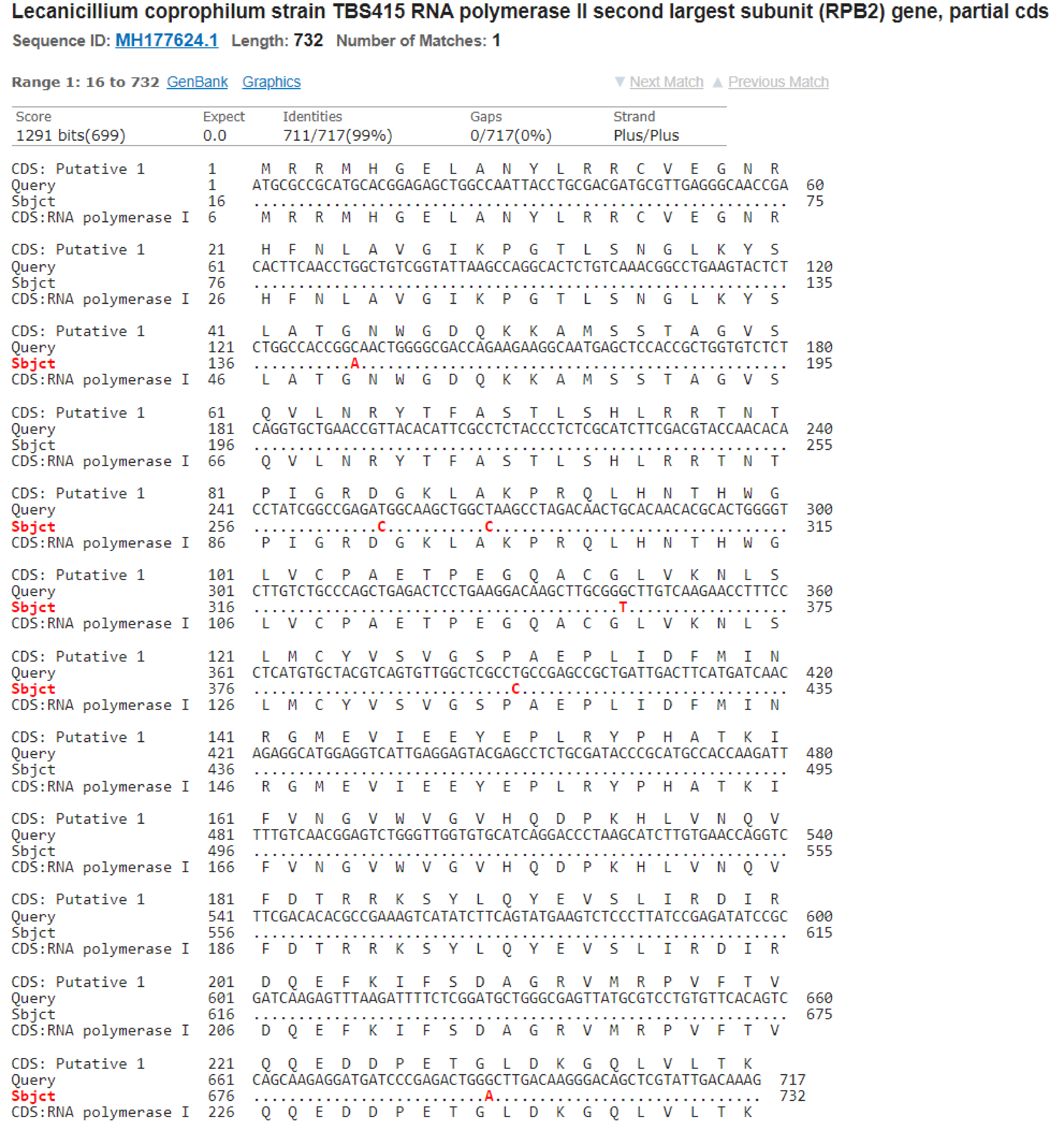

Supplement: Supplementary file 1 — Supplementary Figure S1. [file 41598_2023_43951_MOESM1_ESM.tif]
