# Supplementary material for: First report of emerging fungal pathogens of Cordyceps militaris in Vietnam
Source: Sci Rep. 2023 Oct 17;13:17669. doi: 10.1038/s41598-023-43951-9 (PMC10582018; doi:10.1038/s41598-023-43951-9)
Supplement: Supplementary file 3 — Supplementary Legends. [file 41598_2023_43951_MOESM3_ESM.docx]

**Supplementary Figures**

**Figure S1.** Nucleotide and protein sequence alignment between the *rbp2* marker of strain WF2611 and *Lecanicillium coprophilum* TBS415 (MH177624).

**Figure S2.** Nucleotide and protein sequence alignment between the *rbp2* marker of strain NT1504 and *Calcarisporium coprophilum* CGMCC 3.17940 (KX442607).
